# Clinical, Diagnostic, and Intervention Approach on Yolk Sac Tumor: A Case Report

**DOI:** 10.7759/cureus.71184

**Published:** 2024-10-10

**Authors:** Anjanee Pathak, Nikita Vijay, Anuja Bhalerao

**Affiliations:** 1 Department of Obstetrics and Gynaecology, Narendra Kumar Prasadrao Salve Institute of Medical Sciences and Research Centre, Nagpur, IND

**Keywords:** bilateral salpingectomy, chemotherapy, germ cell tumor, oophorectomy, ovarian neoplasm, staging laparotomy, yolk sac tumor

## Abstract

Endodermal primitive tumors, also known as yolk sac tumors (YSTs), are rare tumors that usually develop in the gonads and are more common in females at premenarchal age. Thus, this report details a rare case of YST in a 13-year-old girl who presented to the Obstetrics and Gynecology outpatient department with complaints of pain and a mass in the abdominal region that had been gradually growing in size for two to three months. The patient also reported a history of weight loss, loss of appetite, intermittent fever, and frequent urination. Per abdomen examination resulted in a mass corresponding to 24 weeks which was firm or hard in consistency with restricted mobility, arising from the pelvis and having an irregular surface. Per rectal examination also reported the same mass on the right side. For investigation magnetic resonance imaging (MRI) was performed that stated the lesion with neoplastic etiology likely arising from the uterus. The treatment plan included a staging laparotomy with a right oophorectomy, bilateral salpingectomy, dissection of the right preaortic and pelvic lymph nodes, and infracolic omentectomy, followed by a course of chemotherapy. The diagnosis of YST was confirmed based on the intraoperative findings and the frozen section report. As this is a rare tumor gynecologists and pediatric physicians should still be aware of this tumor since it can be life-threatening and should be promptly prevented and treated.

## Introduction

Ovarian germ cell tumors (OGCTs) comprise around 15-20% of all ovarian neoplasms, where neoplasms develop in the ovary’s germ cells [1]. Only about 1-2% of OGCTs are categorized as malignant ovarian germ cell tumors (MOGCTs), which make up 3-5% of all malignant ovarian neoplasms [2]. The endodermal primitive tumor, also known as yolk sac tumor (YST) is rare and is the second most frequent type of tumor in MOGCTs [3] that typically occurs in gonads [4]. About 33% of YST patients are premenarchal, and the condition typically affects young women or adolescent girls between the ages of 18 and 30 [4,5]. When compared to epithelial ovarian tumors, YST is a much more malignant tumor that rapidly grows, causes symptoms to subside quickly, metastasizes fast, and invades retroperitoneal lymph nodes and all intra-abdominal structures [6,7].

Before the origin of combination chemotherapy, YST was considered a life-threatening condition. By the end of the 1970s, new chemotherapy regimens had been developed, and as a result, the 5-year survival rates of YST had increased from 14% to almost 90% [4]. Extragonadal sources include the vulva, vagina, cervix, endometrial, sacrococcygeal region, pelvis, retroperitoneum, mediastinum, and other locations. These origins account for about one-third of YSTs [4,8]. Extragonadal YSTs are difficult to diagnose because the disease's signs and symptoms might vary depending on the origin sites [9-11]. Hence, this report describes a rare case of YST in a 13-year-old girl.

## Case presentation

Patient information

A 13-year-old girl reported to the Obstetrics and Gynecology outpatient department with primary complaints of abdominal pain and a mass growing gradually in size for two to three months. Additionally, a history of loss of appetite and weight, fever (on and off) along with frequent urination for 2-3 months was reported by the patient. The menstrual history of the patient described that the patient had not attained menarche yet. Moreover, the patient reported no significant family or past history.

Clinical examination

The patient had stable vital signs but demonstrated pallor and bilateral breast bud. The systemic examination was found to be normal. Per abdomen examination resulted in a mass corresponding to 24 weeks which was firm or hard in consistency with restricted mobility, arising from the pelvis and having an irregular surface. Per rectal examination also reported the same mass more on the right side and the cervix high up. The laboratory investigations revealed serum lactate dehydrogenase (LDH) levels of 656 U/l, carcinoembryonic antigen level of 3.08 ng/ml, CA125 of 171.4 u/ml, beta human chorionic gonadotropin (Bhcg) level of <5.0 ng/ml, and an elevated alpha-fetoprotein (AFP) level of >350 ng/dl.

Diagnostic assessment

**Figure 1:**
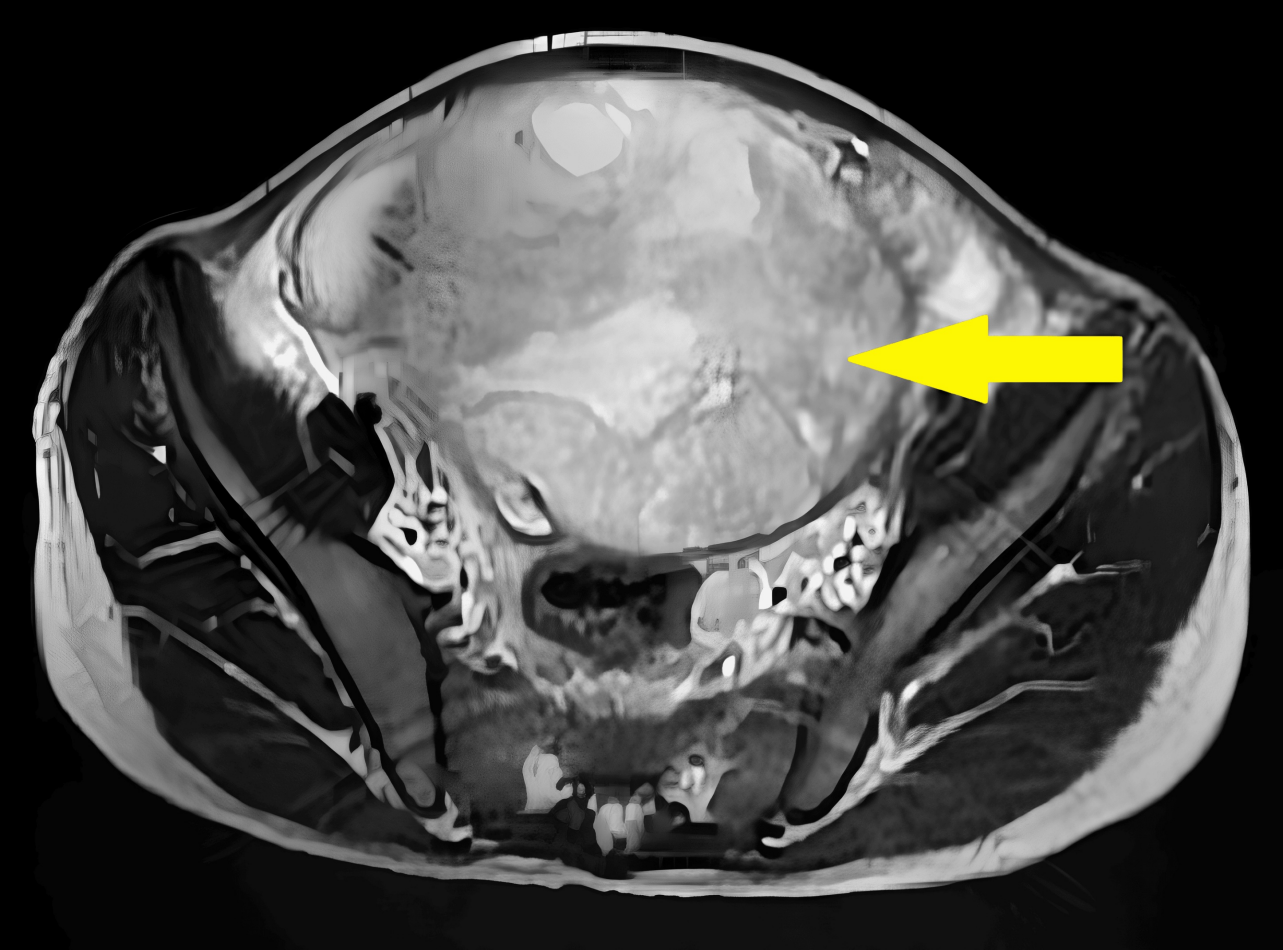
Magnetic resonance imaging (transverse section) illustrating the tumor Yellow arrow = a large well defined lobulated, pelvico-abdominal, soli-cystic altered signal intensity heterogeneously enhancing lesion in the midline extending from S2 to L3 vertebral level.

For further investigations diagnostic assessment consisting of magnetic resonance imaging (MRI) reported a large well defined lobulated, pelvico-abdominal, solid-cystic altered signal intensity heterogeneously enhancing lesion in the midline extending from S2 to L3 vertebral level with few areas of diffusion restriction possibly in continuation with the vagina as illustrated in Figure 1. Bilateral ovaries were not appreciated. Imaging findings were suggestive of neoplastic etiology likely arising from the uterus and therefore, the diagnosis of ovarian neoplasm was confirmed.

Therapeutic intervention

**Figure 2:**
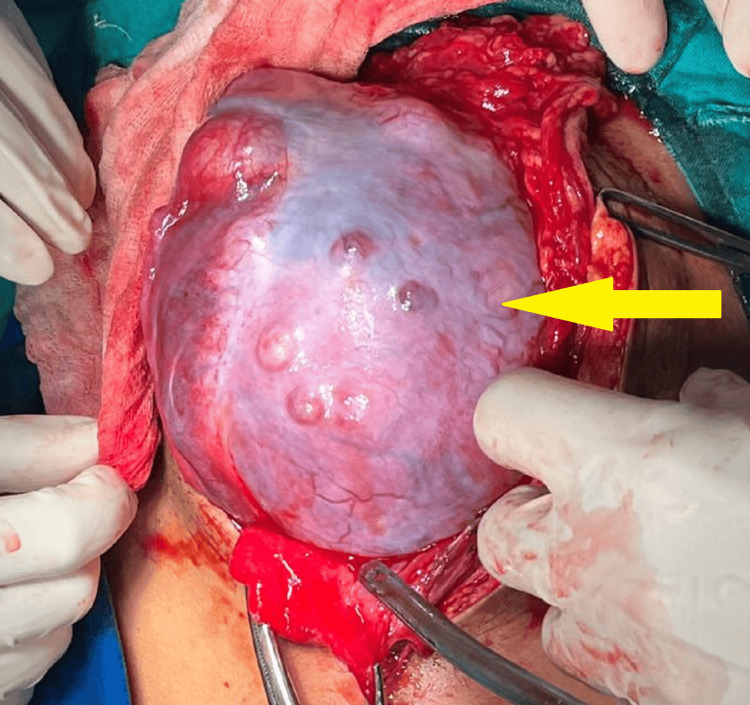
Vascular, adherent mass arising from the right ovary Yellow arrow = yolk sac tumor of stage III A1

The patient was advised for staging laparotomy which involved right oophorectomy with bilateral salpingectomy with right pelvic and preaortic lymph node dissection with infracolic omentectomy. Under all aseptic precautions, under general anesthesia with the patient in a supine lying position, the procedure was commenced. A midline vertical incision extending from the pubic symphysis up to the xiphisternum was made. The abdomen was opened in layers up to the peritoneum and peritoneal fluid was observed and was sent for cytology. A vascular, adherent mass of approximately 15×12×10 cm was noted arising from the right ovary as illustrated in Figure 2.

**Figure 3:**
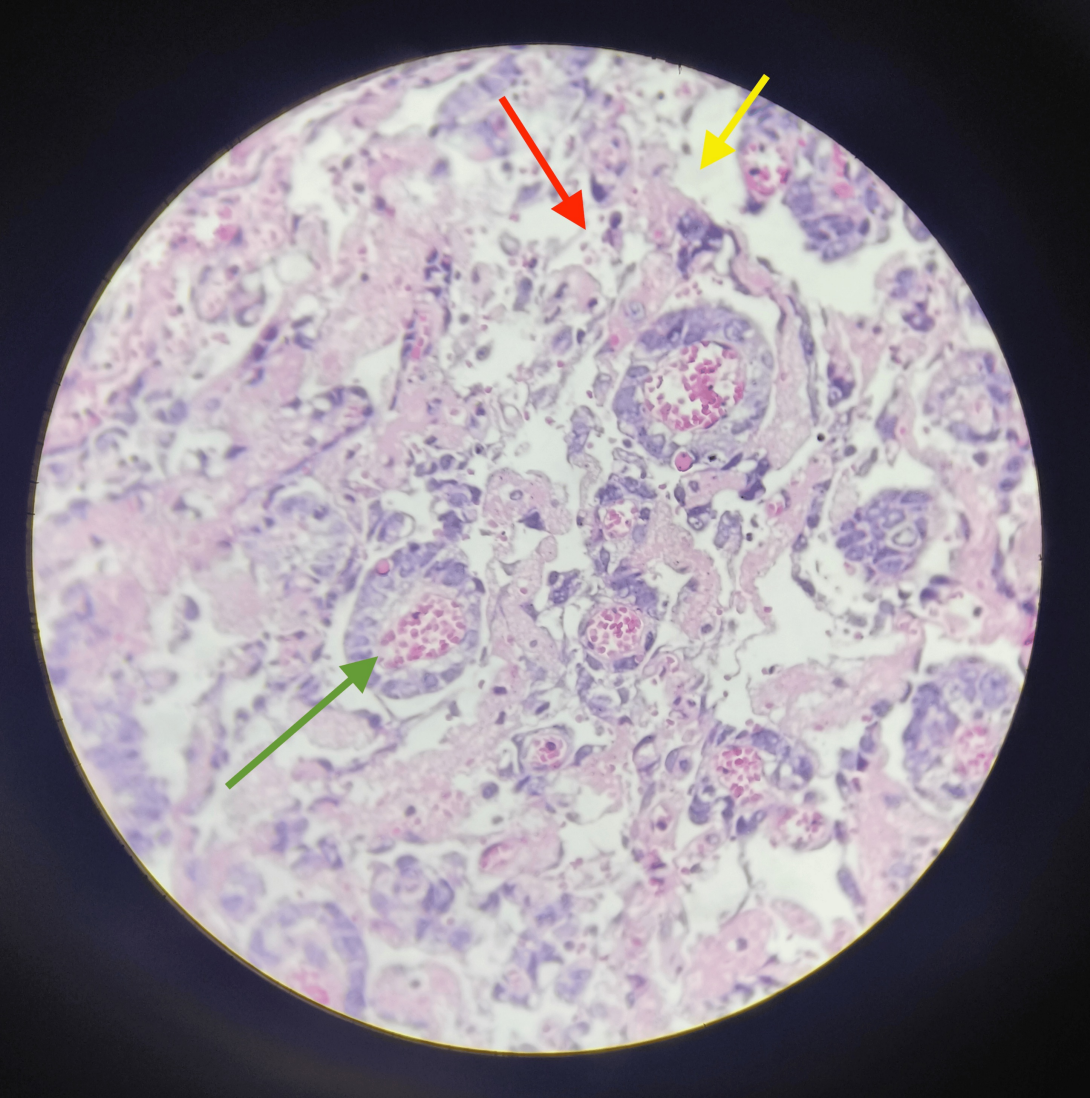
Histopathological examination illustrating yolk sac tumor Yellow arrow = microcystic space, red arrow = loose reticular pattern, green arrow = Schiller-Duval bodies

Multiple adhesions were seen over the omentum, intestines, and uterus. The uterovesical fold was separated and the bladder was pushed down. Resection of the margins of the ovarian mass was performed systematically using a clamp, cut, ligate technique and cautery. The right round ligament was clamped cut and transfixed. The right fallopian tube along with the ovarian mass was removed and sent for histopathological examination (HPE) and frozen biopsy. Tissue noted to be adhered to the posterior wall of the uterus was resected gently using cautery and saline irrigation. Uterus left intact in situ, noted to be hypoplastic. The left ovarian margin cleared and the left ovary was left intact in situ. The left round ligament was clamped cut and transfixed. The left-sided fallopian tube was removed and sent for HPE. The contralateral fallopian tube was removed as it was badly adhered to the posterior surface of the uterus. Intraoperative surgical assistance for dissection between the sigmoid colon and the tumor was done by the surgeon. Three serosal tears of 1×1 cm were noted that were sutured back using vicryl 3-0. Hemostasis was achieved. The frozen section report confirmed germ cell tumor diagnosis consisting of YST of stage III A1 according to the International Federation of Gynaecology and Obstetrics (FIGO) staging as illustrated in Figure 3. Based on the report, the decision for further staging was taken.

Infracolic omentectomy was done, and right-sided common and internal iliac lymph nodes were resected. The abdominal cavity was examined and saline wash was given and suctioned. Hemostasis was achieved. Bilateral pelvic wall, peritoneal, and bladder reflection biopsies were taken and sent for HPE. The abdomen was closed in layers using a loop proline. Skin closed using ethilon and stapler pins intermittently. A hemostatic dressing was performed and the patient was extubated and shifted to the pediatric intensive care unit (PICU). The patient received antibiotics and analgesics. Following an uneventful postoperative phase, the patient was referred for chemotherapy under strict follow-up. The chemotherapy plan consisted of a bleomycin, Etoposide, and cisplatin (BEP) regimen which involved a total of four cycles that were performed as one cycle per week for four consecutive weeks.

## Discussion

In the current case, a 13-year-old girl was diagnosed with a GCT consisting of YST. For diagnostic purposes, an MRI was performed. The management consisted of staging laparotomy and the frozen section report confirmed the diagnosis of YST. MOGCTs can be germinomatous and non-germinomatous and the most prevalent non-germinomatous MOGCTs are YST. Compared to 35% of YST in adults, the great majority of YST (85%) presented as clinical stage I in children [4,12].

The precise pathology of YST is yet unknown. Nonetheless, certain research suggests that it arises from the malignant conversion of misdirected germ cells. GCTs can develop anywhere along the migration from the mesoderm to the future cranial region as germ cells migrate laterally between the embryonic ectoderm and endoderm during the fourth and sixth week of embryogenesis [4,13,14]. Despite the lack of clarity surrounding the pathophysiology of extragonadal GCTs, two theories have been put forth: 1) during embryonic development misintegration of germ cells, and 2) germ cell dispersion to other organs [4]. Therefore, to provide an effective treatment for YST, further research is needed to investigate the pathogenic mechanism.

A similar case was observed in a 9-month-old female in which the tumor was of size 10×10×7.4 cm and was located at the right lower chest and upper abdomen and intervention was provided through chemotherapy [15]. Additionally, in a previous case, a 13-year-old girl had a tumor that measured 14.3 x 14.5 x 8.0 cm and had displaced the front of the uterus. Four sessions of chemotherapy were administered after a unilateral salpingo-oophorectomy and partial omentectomy [16]. Many diagnostic techniques, including ultrasound (US), computed tomography (CT), MRI, and HPE, are used in YST. Moreover, to determine further details related to the tumor, surrounding structures involved, and biopsy exploratory laparotomy is emerging as a more common procedure [17]. Hence, in the present case, all the tests performed confirmed the diagnosis.

The surgical intervention that involved staging laparotomy consisted of right oophorectomy with bilateral salpingectomy with right pelvic and preaortic lymph node dissection with infracolic omentectomy. On the other hand, prior research suggested that surgical procedures include bilateral oophorectomy, peritoneal cleaning, omental biopsy, and selective excision of swollen lymph nodes. An open technique should be used to remove the afflicted ovary completely, preserving its intact tumor capsule as opposed to breaking or rupturing it and biopsy of a normal contralateral ovary is not recommended [18,19]. Reserving fertility through the use of a fertility-sparing technique is an important consideration when treating young patients. Because most tumors are unilateral, it is possible to do fertility-sparing surgery. It has also been demonstrated that minimally invasive surgery has a better prognosis [12].

The current standard of care for YST is first surgery followed by adjuvant chemotherapy with BEP. For primary, metastatic, or recurring disease, BEP seems to be the most effective active first-line treatment when compared to other regimens. Chemotherapy's effects on gonadal and reproductive function are a further crucial factor. Nowadays, the goal of chemotherapy for early-stage diseases is to maximize effectiveness while simultaneously retaining reproductive function and minimizing toxicity [12]. Therefore, even though the majority of patients, especially in the early stages, will maintain their ovarian function and fertility, the possibility of infertility following YST treatment is always a concern.

## Conclusions

The present case highlighted the importance of rare YST in children and provided the findings of various diagnostic tests and approaches related to the surgical intervention and chemotherapy regimen performed as in these patients’ preservation of fertility is an important factor. The usual treatment plan consists of fertility-preserving procedures combined with adjuvant chemotherapy providing a good prognosis in young patients. Even though YST is uncommon in children, pediatricians still need to be aware of this since it can be life-threatening and should be promptly prevented and treated.
